# The Dataset of Daily Air Quality for the Years 2013-2023 in Italy

**DOI:** 10.1038/s41597-026-07712-3

**Published:** 2026-07-07

**Authors:** Alessandro Fusta Moro, Alessandro Fassó, Jacopo Rodeschini

**Affiliations:** 1 Department of Engineering and Applied Science, University of Bergamo, Dalmine, Italy; 2 Department of Economics, University of Bergamo, Bergamo, Italy

## Abstract

Air quality and climate are major issues in Italian society and lie at the intersection of many research fields, including public health and policy planning. There is an increasing need for readily available, easily accessible, ready-to-use and well-documented datasets on air quality and climate. In this paper, we present the GRINS_AQCLIM dataset^1^, created under the GRINS project framework, covering the entire Italian domain for the period 2013-2023. It includes daily statistics (e.g., minimum, quartiles, mean, median and maximum) for a collection of air pollutant concentrations and climate variables at the locations of the 744 available monitoring stations. Input data are retrieved from the European Environmental Agency and Copernicus Programme and were subjected to multiple processing steps to ensure their reliability and quality. Datasets are hosted on Zenodo under open-access principles.

## Background & Summary

In this paper, we present a new dataset for air quality in Italy called “GRINS_AQCLIM_points_Italy”, short “GRINS_AQCLIM”, created under the framework of the Growing Resiliant INclusive and Sustainable (GRINS) project, funded by the NextGeneration EU.

The GRINS_AQCLIM dataset^1^ is based on the measurements of air pollutant concentrations recorded by the Italian ground-based monitoring network managed by the Regional Agencies for the Environmental Protection (ARPAs). The dataset provides daily summary statistics at 744 locations over the 11-year period from 2013 to 2023. The airborne pollutants considered are: nitrogen monoxide and dioxide (NO and NO_2_), particulate matter (PM_10_ and PM_2.5_), ozone (O_3_), ammonia (NH_3_), carbon monoxide (CO), and sulphur dioxide (SO_2_), along with various climate variables useful for modelling purpose (i.e., the height of the atmosphere boundary layer, vegetation indexes, relative humidity, temperature, solar radiation, precipitation, wind speed and direction).

### Italian Air Quality and Data Availability

The need for a dataset on air quality in Italy is driven by the high levels of air pollution, especially in some areas, such as the Po Valley (see e.g.^2^). Air quality data have been widely used across numerous scientific contexts. In Italy, these data have been extensively utilised to investigate health impacts, including studies on pregnancy outcomes^3^, COVID-19 mortality (e.g.^4,5^), ruptured abdominal aneurysms^6^, pulmonary fibrosis^7^, and neurodegenerative conditions such as dementia and Parkinson’s disease^8^, among numerous other applications. Furthermore, air quality data have been used as crucial inputs for scenario analyses concerning green urban planning and city management (e.g.^9–11^), as well as for evaluating emission reduction strategies of intensive farming^12^. Trend analysis is an excellent tool for evaluating the effectiveness of such reduction strategies in relation to emission trends and is performed on long-term monitoring datasets^13^, combined, where available, with modelling simulation data^14^. Moreover, air quality data are used for validation of modelling applications (e.g.^15^) and present a significant opportunity for fair comparison of different spatio-temporal statistical models (e.g.^16^).

In recent years, several initiatives have emerged to provide readily accessible air quality datasets for Italy. Notably, the Agrimonia project^17^ focused on correlating particulate matter concentrations with livestock activities and published a daily dataset on Zenodo (https://zenodo.org/records/7956006) covering Lombardy from 2016 to 2021, which has garnered over 6,000 downloads. Concurrently to ground-based monitoring stations, the European Copernicus programme offers hourly air pollutant concentration estimates on a 0. 1° × 0. 1° grid across Europe, derived from an ensemble of Chemical Transport Models^18,19^. Nevertheless, these data frequently exhibit limitations in capturing high peaks^20,21^. Within Italy, ISPRA systematically publishes yearly summary statistics for the main pollutants, i.e. NO_2_, O_3_, PM_10_ and PM_2.5_, including threshold exceedances and quantiles, for individual monitoring stations, on its official website, encompassing a comprehensive period from 2002 to 2022 (https://www.isprambiente.gov.it/it/banche-dati/banche-dati-folder/aria/qualita-dellaria). Beyond the European context, readily available datasets for various air pollutants in China are also hosted on Zenodo^22,23^. Addressing the existing fragmented landscape, the proposed GRINS_AQCLIM aims to standardise access to Italian air quality data initially for the 2013-2023 period, with the prospect of extending both backward and forward in time.

The GRINS_AQCLIM dataset^1^ contains air pollutant levels recorded by the monitoring stations, managed by the regional agencies for environmental protection (ARPAs). Observed data are submitted from the ARPAs to the European Environmental Agency (EEA) through the air quality portal (https://aqportal.discomap.eea.europa.eu), where air quality data for all of Europe can be accessed. Currently, direct access to Italian air quality data is available via regional ARPAs’ websites and the European Environment Agency (EEA) air quality portal. Obtaining comprehensive raw data from regional ARPAs often proves challenging due to incomplete datasets across regions, missing periods, and a lack of API services for automated retrieval. While the EEA offers API access, its raw data frequently necessitate extensive quality control. Common issues encountered within EEA raw files include duplicate station entries across different files, inconsistent temporal resolutions for the same station (e.g., hourly and daily within the same dataset), internal temporal resolution shifts, and overlapping bi-hourly and hourly records. Consequently, data acquired from the EEA need pre-use transformations and validation, as demonstrated in existing research (e.g.^17,24^). These essential quality assurance procedures often involve statistical steps, such as anomaly detection and removal, or missing-data imputation. Transformations typically involve temporal resolution harmonisation.

The lack of a readily available, freely accessible, and thoroughly quality-controlled air quality dataset for Italy represents a significant barrier for researchers. To address this gap, the GRINS AQCLIM dataset^1^ provides a standardised, well-documented, and ready-to-use resource that overcomes common challenges related to data preprocessing and validation. It offers harmonised observations from monitoring stations with extensive spatial and temporal coverage, supported by transparent quality control procedures and uncertainty-aware aggregation methods. In addition, the dataset integrates curated metadata and climate variables aligned with air quality observations, enhancing its usability for interdisciplinary applications.

Potential limitations of the presented dataset include preferential sampling, the unbalanced spatial distribution of monitoring stations, and the choice of areal climate data. In particular, the monitoring network is designed to monitor air pollution in areas of specific interest and therefore does not necessarily follow the principles of representative spatial sampling over the entire domain. Furthermore, the monitoring network does not uniformly cover Italy, with a greater concentration of stations located in Northern Italy. Finally, climate data may alternatively be retrieved from national downscaled products instead of ERA5 data, potentially providing a more accurate representation of local conditions.

### Dataset Overview

**Fig. 1:**
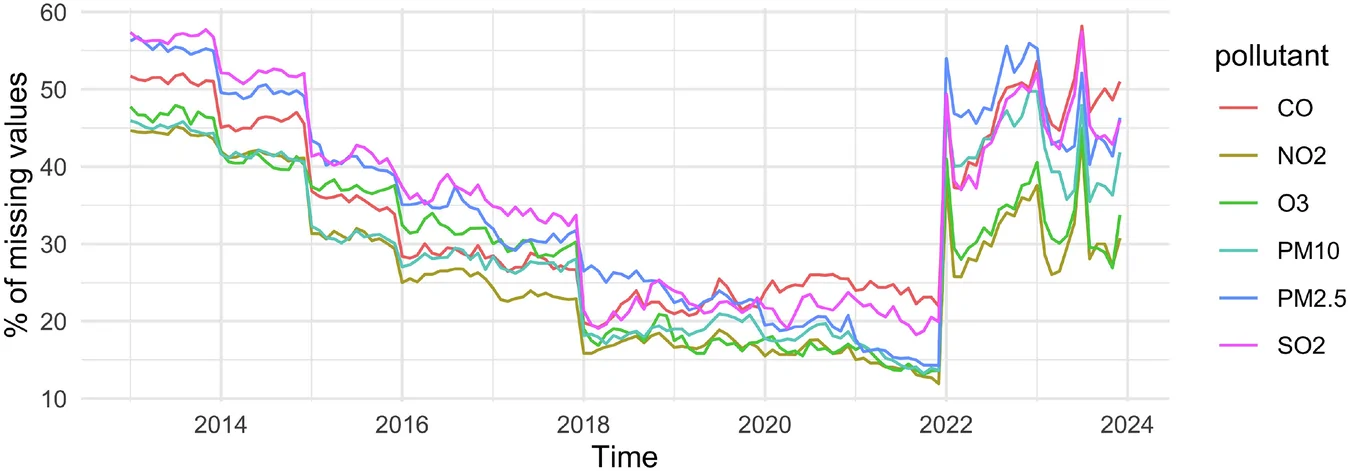
Data availability over time. On the y-axis the percentage of missing values calculated only on the active station for each pollutant.

In Italy, more than 700 land monitoring stations have been working during the last decades. The total number may vary over time as old stations are dismantled and new ones are built. Each station is equipped with various sensors, enabling the measurement of different air pollutants. The GRINS_AQCLIM dataset^1^ comprises data from 744 monitoring stations over 4,017 days, yielding approximately three million rows. Table 1 provides the list of air pollutants included in the dataset, detailing the number of stations that have recorded at least one day of data for each pollutant, alongside their overall minimum, mean, and maximum values, as well as the percentage of missing observations after data cleaning. NO_2_ and PM_10_ exhibit the highest monitoring coverage in Italy, with a large number of active stations (710 and 648, respectively) and relatively low fractions of missing data. The proportion of missing values is computed considering only the active stations for each pollutant and is generally around 30–40%, with higher values observed for NH_3_ and NO. Fig. 1 illustrates the temporal dynamics of data availability on a monthly basis. For clarity, NH_3_ and NO are not shown. The temporal patterns suggest a gradual increase in data availability during the first nine years, especially every two years, followed by a noticeable decrease in the last two years. At the time of data download, i.e. late 2024, some data could still be under review and therefore not available.

**Table 1:** Overview of the air quality (AQ) variables in the GRINS_AQCLIM after data cleaning and temporal transformation. Min, Mean, and Max are expressed as *μgm*^−3^, except for CO that is *mg* *m*^−3^.

Air pollutant	# stations	Min	Mean	Max	Missing (%)
CO	268	0	0.50	58.20	34
NH_3_	1	0	5.71	17.26	57
NO_2_	710	0	21.71	362.21	27
NO	336	0	11.44	583.46	65
O_3_	402	0	56.45	756.33	29
PM_10_	648	0	24.08	2, 575	30
PM_2.5_	351	0	15.51	907	35
SO_2_	274	0	3.11	868.42	36

**Fig. 2:**
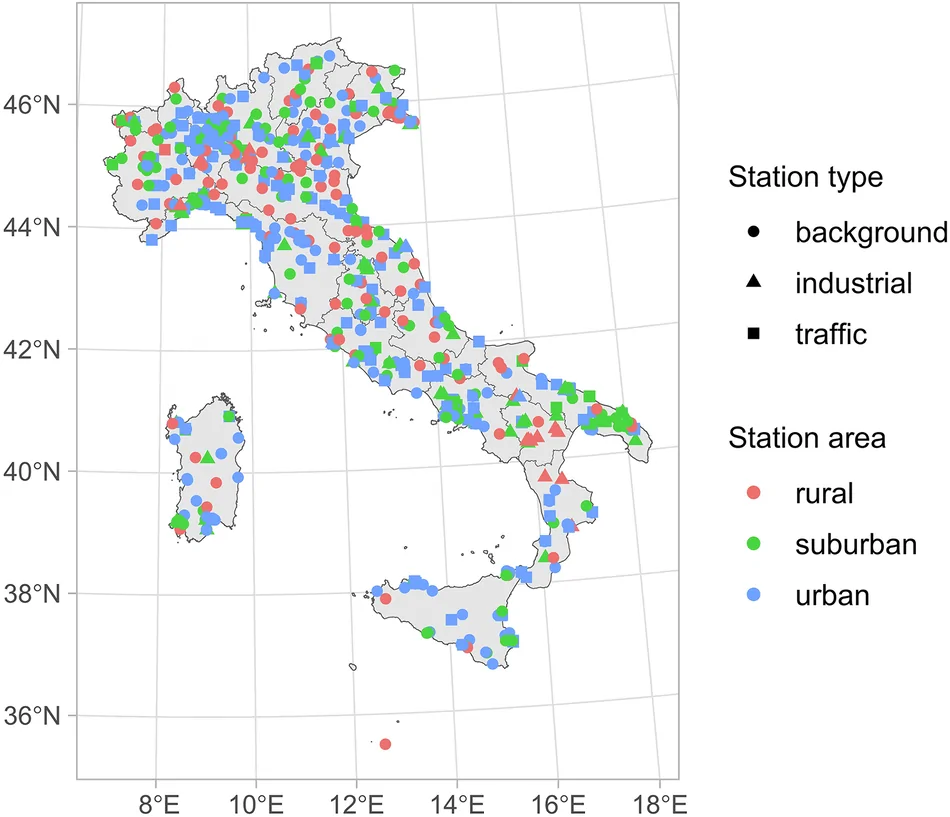
Spatial localisation of the air quality monitoring network in the GRINS_AQCLIM dataset. Station type refers to the predominant emission sources, while Station area indicates the prevalent surrounding land-use context.

Figure 2 illustrates the geographical distribution of air quality monitoring stations within the GRINS_AQCLIM, classified by station type and area in accordance with European classification standards. In particular, station type refers to the predominant emission sources (Background, Industrial, Traffic), while station area indicates the prevalent surrounding land-use context (Rural, Suburban, Urban). Table 2 provides the station distribution by type and area.

**Table 2:** Number of stations by station-type and station-area in the GRINS_AQCLIM dataset.

	Rural	Suburban	Urban	Total
Background	108	120	216	444
Industrial	15	72	11	98
Traffic	1	20	181	202
Total	124	212	408	744

In addition to air pollutant concentrations, the dataset includes relevant climate information to facilitate modelling applications. It is widely known that atmospheric conditions can heavily impact the air quality and, therefore, climate variables are often considered along with airborne concentrations (see e.g.^12,16,25–28^).

## Methods

### Input data

Air quality data are retrieved from the Air Quality Portal - Download Service (https://eeadmz1-downloads-webapp.azurewebsites.net) managed by the European Environmental Agency (EEA). The portal allows manual or API based download. Air pollutants considered are listed in Table 1. The download of 11 years of data required about 2 hours of time, parallelising the request on 50 workers. It resulted in more than 20k CSV files, each containing a single station for a year and a pollutant.

**Fig. 3:**
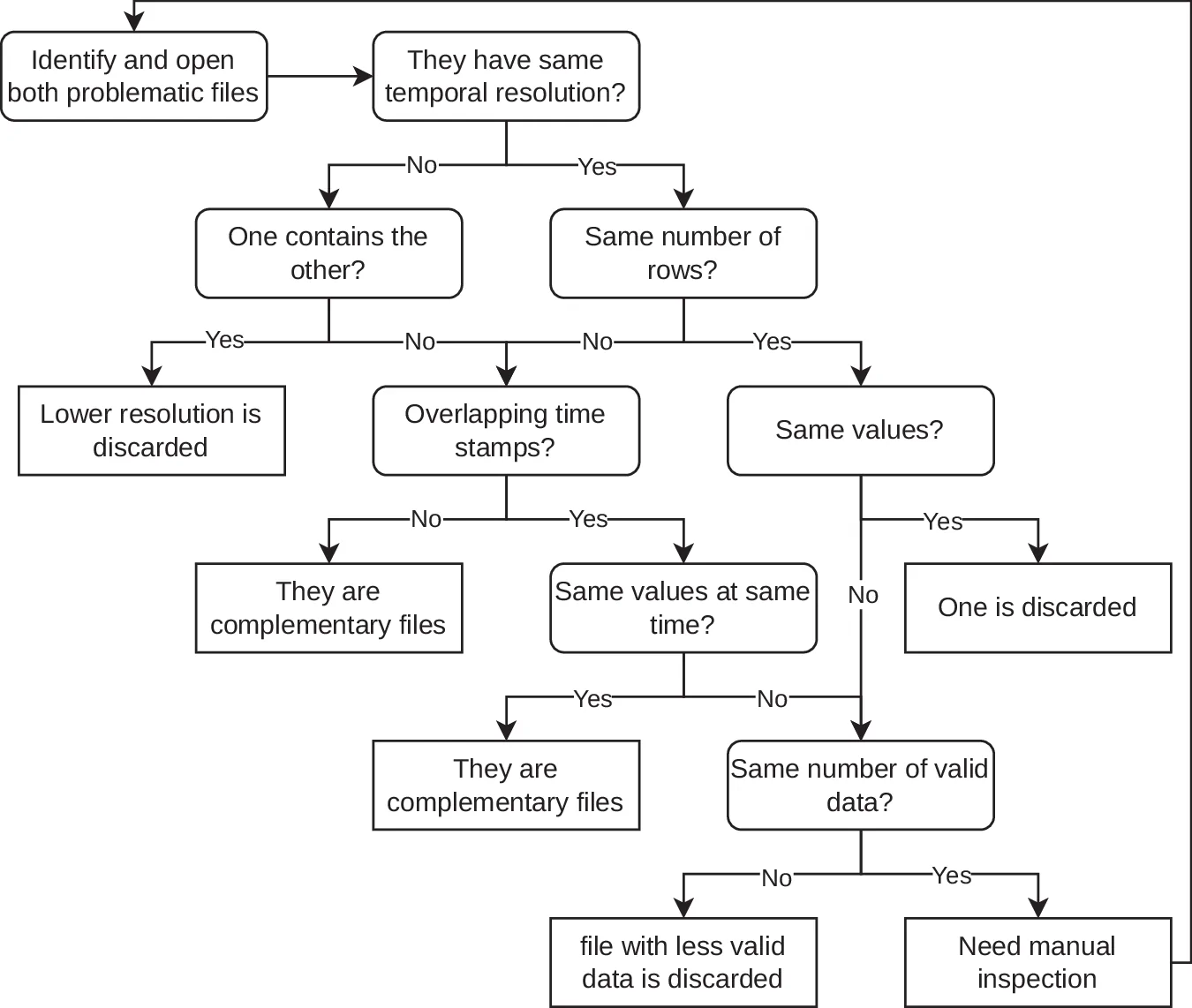
Flow diagram for the automatic fix of duplication issues in raw air quality files from the EEA.

Challenges within the above raw files include duplicated measurements (i.e., identical hourly readings) and instances where two distinct raw CSV files present the same station, pollutant, and period. The number of problematic files exceeds 300. The automated procedure illustrated in Fig. 3 was therefore implemented to resolve the duplication issues of such a large volume of raw files. Ultimately, no files required manual inspection.

The meteorological data are based on the ERA5 datasets^29^ accessed via the Climate Data Store portal provided by the Copernicus Climate Change Service (https://cds.climate.copernicus.eu/datasets/reanalysis-era5-single-levels?tab=overview). These datasets contain the numerical model outputs computed by the European Centre for Medium-Range Weather Forecasts (ECMWF). ERA5 is the fifth-generation ECMWF reanalysis of the global climate for the past decades. The reanalysis combines model data with observations from across the world into a globally complete and consistent dataset using the laws of atmospheric science. The first ERA5 dataset used, called “ERA5-single-level”, provides hourly estimates for various atmospheric and land-surface quantities with a regular grid scheme at various atmosphere levels on a regular latitude/longitude grid of 0.25° × 0.25°. The second ERA5 dataset used, called “ERA5Land, is obtained by downscaling the ERA5 fields^30^ and includes a narrower subset of land-related variables with respect to ERA5, but on a higher spatial resolution (i.e. 0. 1° × 0. 1°). In our study, the ERA5Land dataset was used as the primary source, and ERA5-single-level was employed to fill gaps in areas where ERA5-Land data are unavailable. In fact, ERA5Land variables are not available for marine regions, resulting in missing values for some stations along coastlines or on small islands.

The climate variables retrieved are reported in Table 5 and represent external drivers that typically influence airborne pollutants. These variables are often included as covariates in air-quality modelling studies (e.g.^16,27^). In addition to atmospheric conditions, we also retrieved land-surface variables, specifically vegetation cover, distinguishing between high and low vegetation types.

### Quality check and data cleaning

#### Anomalies handling

After resolving conflicts in the raw files, as explained above, the quality of the air pollution data was examined. The EEA provides a validity flag and a verification flag for each measurement. The validity flag indicates the following status: −99 for data invalidated by station maintenance or calibration; -1 for generally invalid data; 1 for valid data; 2 for valid data below the detection limit (BDL); and 3 for valid BDL data replaced by 0.5 times the detection limit. For this work, an initial filter was applied, setting concentrations with a validity flag of -99 or -1 to the missing value.

Despite this preliminary filtering, anomalous concentration values persisted, including extraordinarily high concentrations (e.g., millions of micrograms per cubic metre for particulate matter). An analysis was therefore performed to exclude these values, which likely stemmed from measurement errors, whether technical or operational.

To identify and exclude anomalous values, various thresholds were investigated. Initially, the verification flag was considered. This flag can assume three values: 1 for verified data, 2 for preliminary verified, and 3 for unverified data. The distribution for each pollutant was analysed conditioned on this flag, expecting that anomalous concentrations would primarily fall into the unverified category. However, this was not the case; extremely high values were distributed across categories (for example, Table 3 shows that the largest value is obtained by the “verified case for SO_2_).

**Table 3:** Summary Statistics for SO_2_ concentrations [*μgm*^−3^] by verification flag category.

Verification Flag	Min	Mean	Max	Sd	Counts (%)
Verified (1)	0.0	3.5	1.4e+06	440	84
Preliminary Verified (2)	0.01	3.7	340	3.3	2
Not Verified (3)	0.0	3.1	2.6e+03	10	14
Overall	0.0	3.5	1.4e+06	400	100

To identify appropriate thresholds, multiples of the standard deviation (e.g. 4 *σ*) were applied to the overall distribution (2013–2023, all stations) of each pollutant. This z-score method, however, assumes a Gaussian distribution, a condition often not met by air quality concentration distributions, which frequently exhibit skewness with a long right tail. To mitigate this, the same procedure was applied to logarithmically transformed data. Nevertheless, this alternative approach yielded inconsistent results across pollutants: for instance, the percentage of particulate matter discarded using 6 *σ* on log-transformed data was significantly lower than that for NO_2_, whereas the opposite occurred when applying the method to untransformed data. The corresponding results are reported in Table 2.5.2 of the GitHub repository: https://github.com/GRINS-Spoke0-WP2/AQ-EEA/tree/main/v.1.0.0. Consequently, a standard approach for all pollutants could not be established using this method.

Therefore, the percentiles of the overall distribution, in particular the 99%, 99.9%, 99.99% and 99.999%, were investigated as possible thresholds. For each percentile, overall statistics were calculated, including the mean, standard deviation, the absolute threshold value, the number of excluded observations, and the count of affected stations.

**Fig. 4:**
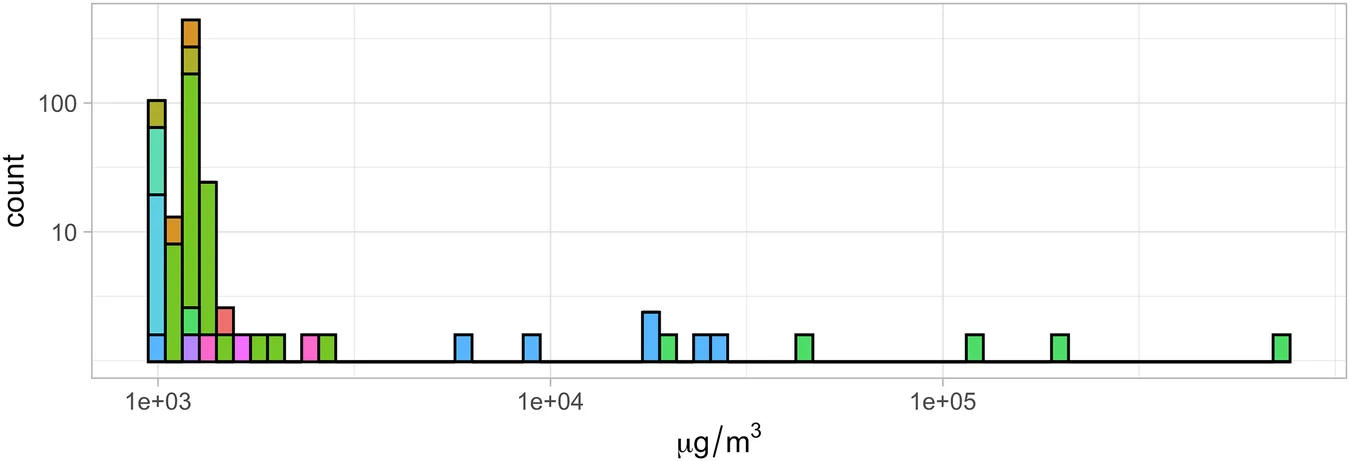
PM2.5 Histograms of observations exceeding the 99.99% percentiles. Colours according to the monitoring station. Blue refers to the station in Alessandria (AL), while green refers to the one in Papariano (UD).

For visual interpretation, histograms of the excluded observations were generated and inspected for each percentile, with data points coloured according to the recording monitoring station. This aimed to identify potential clusters within the same station that might indicate periods affected by anomalous local conditions. Figure 4 displays an example of this visual representation, showing that extremely high PM_2.5_ values (over 10,000 *μgm*^−3^) in the entire dataset were recorded at only two stations, located in Alessandria (AL) and Papariano (UD). These values were deemed attributable to technical problems or anomalous local conditions and were subsequently set as missing values.

In conclusion, by jointly considering the table of percentiles and histograms (available on the GitHub page, table 2.5.3 and plot/preprocessing/anomalies, respectively, at https://github.com/GRINS-Spoke0-WP2/AQ-EEA/tree/main/v.1.0.0), fixed thresholds were established. In particular, the chosen values were defined so as not to truncate the tail of the distributions, ensuring that low-frequency but still meaningful observations (i.e., recorded by different stations) are preserved in the dataset. The thresholds selected, the proportion of values over those thresholds, the corresponding percentile, and the number of stations affected are listed in Table 4. Thresholds are expressed in micrograms per cubic meter, except for CO, which is milligrams per cubic meter. These thresholds were applied throughout the analysis to assess air quality levels across the monitoring network: all exceeding concentration values are set as missing.

**Table 4:** Thresholds identified for each pollutant for quality-control procedures, expressed in *μg* *m*^−3^, except for CO, which is expressed in *mg* *m*^−3^. “Excluded (%)” denotes the proportion of observations exceeding the threshold. “Percentile” refers to the corresponding percentile of the overall distribution, while “# stations” indicates the number of stations affected by over-threshold values.

Air pollutant	Threshold	Excluded (%)	Percentile	# stations
CO	100	2.3e-06	99.99977	1
NO	1000	1.3e-06	99.99987	3
NO2	1000	3.7e-07	99.99996	4
O3	1000	7.0e-07	99.99993	11
PM10	2630	9.8e-06	99.99902	8
PM2.5	980	1.6e-04	99.98385	13
SO2	10e03	1.4e-06	99.99986	1

#### Monitoring Station Registry

An important step within the quality check procedure is the information associated with the monitoring stations. This information encompasses geographical coordinates (longitude and latitude, defining a unique spatial reference point) and the station’s classification according to European standards (i.e., by area type and emission source), among other details. During this process, it was identified that three stations shared identical coordinates despite having distinct names and concentration readings. These discrepancies were manually rectified by cross-referencing metadata provided by the regional agency, which also supplied information regarding the sensors installed at each station. Furthermore, 32 stations were found to be duplicated within the EEA station registry, erroneously assigned to two different area categories simultaneously (e.g., classified as both “traffic and “industrial). To resolve these inconsistencies, the surrounding area for these stations was manually verified using satellite imagery and subsequently corrected.

### Harmonisation of temporal and spatial resolution

Before computing daily summary statistics, we noticed that temporal resolution varies among air quality monitoring stations in the EEA dataset. In particular, although almost all air pollutants are available at the hourly scale, some particulate matter data are already at daily resolution. For hourly data, we imputed hourly missing values to prevent bias in daily summary statistics. Similarly to^17^, this has been done by fitting a local level model^31^ to hourly data for each station and imputing the missing values by the estimates obtained through the Kalman smoother, as discussed in the Technical validation Section.

However, daily values were still designated as missing if more than five consecutive hourly data points were missing. To maximise information retention during the conversion from hourly to daily resolution, the minimum, first quartile, mean, median, third quartile, and maximum values were computed. Median, first and third quartiles are calculated using the R function “quantile” from the “stats” package, setting *p* to 0.5, 0.25 and 0.75 respectively in the function

1 $$Q(p)=(np-p-j){x}_{j}+(np+1-p-j){x}_{j+1}$$

where *x*_*j*_ is the *j*th order statistic of the considered day, *j* is the integer part of (*np* + 1 − *p*),  *n* is the sample size, that is, the number of hourly observations for the specific day.

The hourly climate variables have been converted to the daily resolution using the transformations detailed in Table 5. Moreover, the spatial matching between the air quality monitoring network points and the climate variable grid was performed using the “point-extraction” method. This method consists in associating each monitoring station with the daily climate conditions present within the grid cell to which the station belongs.

**Table 5:** Description of daily climate variables included in the GRINS_AQCLIM.

Variable Name	Description	Unit
CL_lai_hv	Constant fixed value of high vegetation leaf area index	*m*^2^/*m*^2^
CL_lai_lv	Constant fixed value of low vegetation leaf area index	*m*^2^/*m*^2^
CL_rh	Mean of relative humidity	%
CL_ssr	Maximum of surface solar radiation	*J*/*m*^2^
CL_t2m	Mean of temperature at 2 meters	°*C*
CL_tp	Total precipitation	*m*
CL_winddir	Mode of wind direction (1=N, 2=E, 3=S, 4=W)	—
CL_windspeed	Mean of wind speed	*m*/*s*
CL_blh	Mean of the height of the atmosphere boundary layer	*m*

## Data Records

Data can be downloaded from the open-access Zenodo page (https://zenodo.org/records/17605148) and will also be available on the Amelia platform (https://ameliadp.grins.it/datasetGuest). Regarding the GRINS_AQCLIM dataset^1^, the repository is based on three main files containing: the daily statistics on air quality and climate, the information about monitoring stations, and the missing imputation uncertainty propagated to daily statistics. The files are named GRINS_AQCLIM_points_Italy, Station_registry_information and GRINS_AQCLIM_imputation_uncertainty, respectively, and are available in CSV and R format.

Since the GRINS_AQCLIM_points_Italy dataset is divided into two dimensions, Air Quality (AQ) and Climate (CL), the column names have two different prefixes, namely AQ_ and CL_. The dataset includes the “AirQualityStation column, which reports the identification code of the station, and the column “time, which identifies the day. There are 48 columns combining the prefix “AQ_ with summary statistics (i.e. “min, “q1, “mean”, “med, “q3, “max) and the pollutant name. For instance, the column name “AQ_q1_NO2 refers to the first quartile of the daily distribution of nitrogen dioxide recorded by each particular station on each particular day. Considering the climate part, variables included are listed in Table 5, and more information about these variables can be found on the European Climate Data Store webpage (https://cds.climate.copernicus.eu/datasets/reanalysis-era5-single-levels?tab=overview).

The Station_registry_information.csv file contains useful information on the monitoring stations, in particular: the “AirQualityStation field which identifies the name of the station, “Longitude and “Latitude are the coordinates in the reference system EPSG 4326 WGS-84, “Altitude the altitude of the station, “AirQualityStationType the type of prevalent emission sources, “AirQualityStationArea the prevalent type of area surrounded it, as discussed in previous sections. This information does not change over time, so they are contained in a separate file to optimise storage.

The estimated imputation uncertainty for daily statistics is provided in the GRINS_AQCLIM_imputation_uncertainty dataset. These estimates are calculated considering both variances and covariances through all the daily summary statistics. In particular, if the minimum or maximum daily values are imputed, the root square of the associated Kalman smoother conditional variance is reported. For quartiles and median, only conditional variances and covariances of the two selected ordered statistics are considered. For the daily average, all the conditional variances and covariances intra-day are considered. In this case, we notice that zero values refer to daily averages without missing values at the hourly level, missing values refer to missing daily averages, and positive uncertainties are related to daily averages with one or more hourly imputed values. More details about missing-data imputation are available in the Technical validation section. We would like to remind the reader that each row in the GRINS_AQCLIM dataset^1^ corresponds to a specific station and a specific day, and around 47% of these station-day situations did not require imputation. Therefore, to save storage space, we excluded these rows from the GRINS_AQCLIM_imputation_uncertainty dataset.

## Technical Validation

To validate the GRINS_AQCLIM dataset^1^, comparisons were made with established external data sources. To compare the time series from different datasets we used the Root Mean Squared Difference (RMSD), defined as ${\rm{RMSD}}={(1/N{\sum }_{i=1}^{N}{({y}_{i}^{(A)}-{y}_{i}^{(B)})}^{2})}^{\frac{1}{2}}$, where *y*^(*A*)^ and *y*^(*B*)^ denote the time series of datasets *A* and *B*, respectively. Low RMSD values indicate strong consistency between the datasets.

**Fig. 5:**
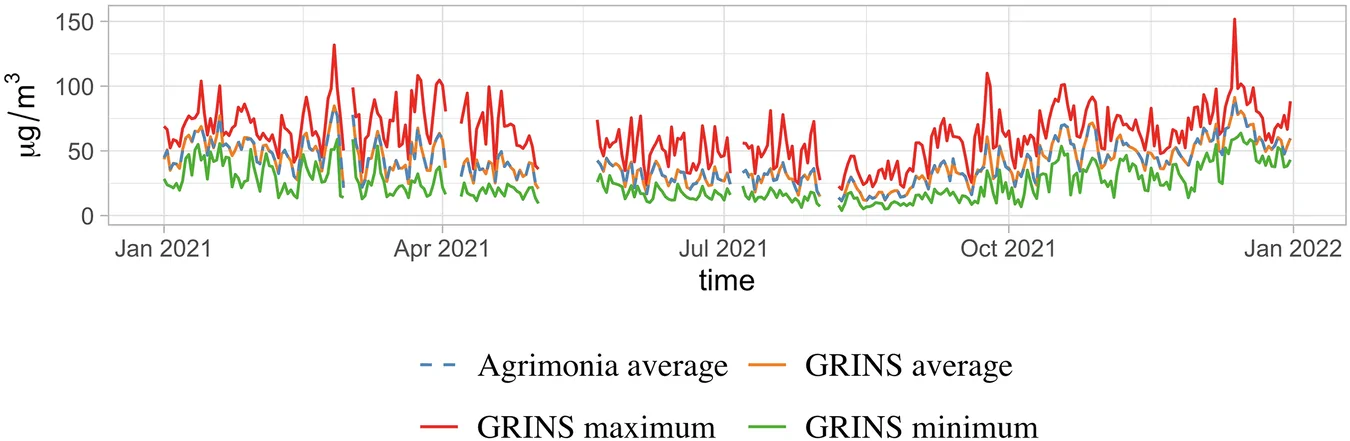
NO_2_: Comparison of nitrogen dioxide concentrations provided by the Agrimonia and GRINS_AQCLIM. Data for Milan city centre. RMSD = 0.8 *μg*/*m*^3^.

Air quality data were compared with the Agrimonia dataset^17^, discussed above. The Agrimonia dataset was specifically chosen because its air quality data are directly obtained from the Lombardy regional agency, rather than from the EEA. Consequently, station names differ, and locations may vary slightly. Based on georeferencing we identified matching stations across the two datasets and computed the RMSD for each pair. Table 6 reports the number of station pairs considered, as well as the minimum, mean, and maximum RMSD values, disaggregated by pollutant. The analysis shows strong consistency between the two datasets, with RMSD values always below 1 *μg* *m*^−3^ and below 0.02 *mg* *m*^−3^ for CO. We also present a visual inspection of this comparison, limited to NO_2_ data for one station pair located in Milan city centre. Figure 5 illustrates the two time series of daily average values, along with the daily minimum and maximum values from the GRINS_AQCLIM dataset^1^, for the year 2021. The time series show strong consistency, with the daily average values from the Agrimonia dataset consistently falling within the range defined by the GRINS_AQCLIM daily minimum and maximum, and almost exactly overlapping with the daily average. The RMSD between the two daily averages is 0.8 *μg*/*m*^3^.

**Table 6:** Station RMSD comparing Agrimonia and GRINS_AQCLIM datasets concentration values.

Pollutant	# stations	Min	Mean	Max
PM_10_	28	0.271	0.288	0.304
PM_2.5_	14	0.271	0.291	0.302
CO	16	0.011	0.013	0.018
NO_2_	41	0.171	0.546	0.957
SO_2_	13	0.010	0.048	0.161

# stations is the number of station pairs compared. Min, Mean and Max refer to the RMSD. Unit of measure *μg* *m*^−3^, except for CO, which is *mg* *m*^−3^. The period considered is the year 2021.

**Fig. 6:**
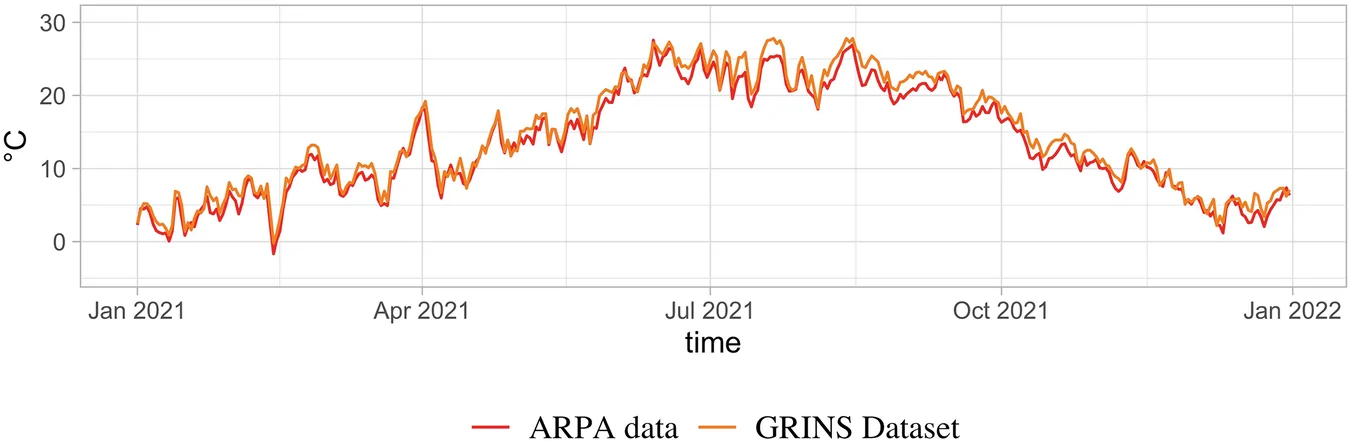
Comparison of temperature data provided by regional agency (from ground-based monitoring station) and data contained in the GRINS_AQCLIM (from ERA5 dataset) for Como city. RMSD = 1.32 °C.

ERA5 datasets are generally regarded as reliable sources for climate data, and extensive validation studies are available in the literature (e.g.^32,33^). For instance, in Italy, ERA5 temperature and precipitation data have shown good consistency with other reanalysis products and high agreement with ground-based observations^34,35^. Our comparison is intended only as a technical validation of the implemented algorithms, rather than a comprehensive assessment of reanalysis performances. We compared temperature data from a ground-based weather monitoring station with the temperature data contained in the GRINS_AQCLIM dataset^1^ for a geographically corresponding air quality monitoring station during the year 2021. In-situ observations were obtained from the regional environmental protection agency (https://www.arpalombardia.it/temi-ambientali/meteo-e-clima/form-richiesta-dati/) for the Como area. Figure 6 shows a good agreement between the two time series, with an RMSD of 1.32°C, supporting the physical consistency of the climate variables in the presented dataset.

### Measurement uncertainty

The air quality data used here are based on reference measurements. This means they are obtained by well-established measurement instruments and procedures, such as the European Parliament’s Directive 2008/50/EC. It is worth noting that European air quality legislation has recently been updated with Directive 2024/2881/EC, which will replace Directive 2008/50/EC following its transposition by Member States by December 2026. However, the present study is based on measurements collected under the previous regulatory framework (Directive 2008/50/EC), which was in force at the time of data acquisition. According to this, the uncertainty (expressed at a 95% confidence level) should not exceed a certain percentage of the corresponding limit value. The maximum percentage is 15% for all pollutants except particulate matters, which have a 25% limit. This must hold for each averaging period considered. Despite this, neither EEA nor local protection agencies provides the uncertainty at the single data level.

ECMWF provides a measure of uncertainty for ERA5, calculated from the ensemble of data assimilations (EDA) system. The EDA accounts primarily for random uncertainties in observations, sea surface temperature and the physical parametrisations of the model. However, systematic model errors are not considered by the EDA. For affordability reasons, the EDA has a lower resolution than ERA5, making it unable to directly describe all of ERA5’s uncertainties. Nevertheless, it can be useful for comparing recent days to past decades or for a given day or season affected by strong weather behaviours, such as areas near tropical cyclones or along storm tracks. Considering the spatial and temporal domain of the dataset, we have decided not to include this measure in the dataset. Uncertainty is available on the ERA5 webpage hosted on CDS, under the category ensemble spread (https://cds.climate.copernicus.eu/datasets/reanalysis-era5-single-levels?tab=download).

### Missing data imputation and associated uncertainty

**Fig. 7:**
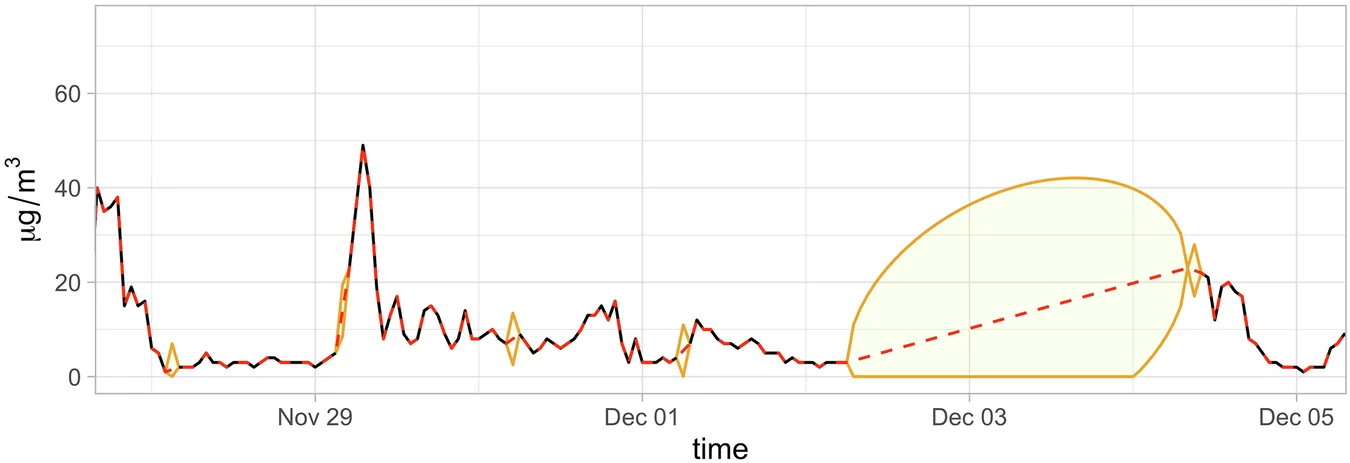
NO_2_: Example of missing imputation procedure for five days and one station measuring NO_2_. Black line is observed data, red dotted line is the imputed values, orange bands represents the standard deviation.

Air quality data from ground-based monitoring stations are affected by missing values due to a variety of possible causes, either technical or manual. To convert hourly data to the daily resolution, missing hourly values were imputed to avoid bias. For each 11-year hourly time series, all missing values were imputed. However, days containing more than five consecutive hours of missing data were recorded as missing in the daily dataset. To impute the missing hourly data we assume that the air quality concentrations follow a random walk, where the variance of the innovation term is estimated through the L-BFGS-B^36^ method. L-BFGS-B is a box-constrained and limited-memory version of the well-known Broyden-Fletcher-Goldfarb-Shanno algorithm (BFGS)^37^. After the estimation of the variance parameter, the Kalman smoother is run in order to estimate the hourly missing values and their uncertainties. An example of this technique is shown in Fig. 7. Clearly, the large variance for the gap 3rd December results in a missing daily value according to the procedure explained above.

Along with daily values we provide uncertainty quantification. The variance of the daily mean depends on the conditional variances and covariances of the imputed values. In particular, considering the day *d* and its 24 hours, the variance of the daily average ${\bar{y}}_{d}$ is calculated as:

2 $$Var({\bar{y}}_{d})=\frac{1}{2{4}^{2}}\left(\sum _{i\in d}Var({y}_{i})+2\sum _{\begin{array}{c}i,j\in d\\ i\ne j\end{array}}Cov({y}_{i},{y}_{j})\right)$$

where *y*_*i*_, *i* ∈ *d*, are the hourly concentrations of day *d*. In our case, the variances of the observed data are set to 0 while the variances and the covariances of imputed values are derived from the Kalman smoother (see e.g.^38^ for more details). In particular, for *n* data and *i* = {1, …, *n*}, assume *P*_*i*_ = *Var*(*y*_*i*_∣*Y*_*i*−1_) is the conditional variance of the Kalman filter at time *i*, and *V*_*i*_ the conditional variance of the Kalman smoother (i.e., *Var*(*y*_*i*_∣*Y*_*n*_)), where ${Y}_{j}={({y}_{1},\ldots ,{y}_{j})}^{T}$. The variances of imputed values are *V*_*i*_, while the covariances are calculated as:

3 $$Cov({y}_{i},{y}_{j})={P}_{i}({L}_{i}\cdots {L}_{i-j})(1-{N}_{j-1}{P}_{j})\quad \,{\rm{for}}\,\quad i,j\in \tau \quad \,{\rm{and}}\,\quad i > j$$

where *τ* ⊆ {1, …, *n*} are the time stamps related to missing observations of day $d,\,{N}_{j-1}={P}_{j}^{-1}-{L}_{j}^{2}{N}_{j}$ is obtained recursively setting *N*_*n*_ = 0. *L*_*j*_ is set to 1 for missing values (i.e. *j* ∈ *τ*) and 0 otherwise. Consequently, the product *L*_*i*_ ⋯ *L*_*i*−*j*_ is different from 0 only for consecutively missing data.

As discussed above, the GRINS_AQCLIM dataset^1^ provides, in addition to the daily mean, the daily minimum, first quartile, median, third quartile, and maximum values. When one of these statistics corresponds to an imputed observation, the associated uncertainty reported in the GRINS_AQCLIM_imputation_uncertainty dataset is the conditional variance of the imputed value estimated through the Kalman smoother.

## Data Availability

Data can be downloaded from the Zenodo page (https://zenodo.org/records/17605148) and they will be accessible through the Amelia platform (https://ameliadp.grins.it/datasetGuest). The repository contains three main datasets: the dataset on air quality, the information about the ground-based monitoring stations and the missing imputation uncertainty propagated to daily statistics. The files are named GRINS_AQCLIM_points_Italy, Station_registry_information and GRINS_AQCLIM_imputation_uncertainty, respectively and are available in CSV and R format. The GRINS_AQCLIM_points_Italy dataset is divided in two dimensions: Air Quality (AQ) and Climate (CL). The names of the columns have two different suffixes, following this division (i.e. AQ_ and CL_). The Station_registry_information dataset contains useful information about the monitoring stations (e.g. georeferentiation). The GRINS_AQCLIM_imputation_uncertainty dataset contains estimated imputation uncertainty for daily statistics.

## Code availability

All the code used to generate the dataset is available at the GitHub page of the GRINS project (https://github.com/GRINS-Spoke0-WP2/AQ-EEA/tree/main/v.1.0.0 for air quality, and https://github.com/GRINS-Spoke0-WP2/WE-C3S for climate), online and freely consultable. Moreover, on the GitHub page there is extensive documentation that explains the R scripts. The software version used is R 24.3.0.
